# Self-Concept and Physical Activity: Differences Between High School and University Students in Spain and Portugal

**DOI:** 10.3389/fpsyg.2019.01333

**Published:** 2019-06-20

**Authors:** Wanesa Onetti-Onetti, José Luis Chinchilla-Minguet, Fernando Manuel Lourenço Martins, Alfonso Castillo-Rodriguez

**Affiliations:** ^1^Faculty of Education, UNIR, International University of La Rioja, Logroño, Spain; ^2^Department of Languages, Arts and Sports, University of Málaga, Málaga, Spain; ^3^Instituto Politécnico de Coimbra, Coimbra, Portugal; ^4^Instituto de Telecomunicações, Covilhã, Portugal; ^5^Department of Physical Education and Sports, University of Granada, Granada, Spain

**Keywords:** physical activity, self-concept, adolescents, educational stage, IPAQ

## Abstract

The period of adolescence stands out as a critical and decisive phase, first because it leaves its mark on personality development, which is affected by psychosocial factors, and second because the healthy lifestyle habits acquired during this stage form a foundation for adulthood. The objective of the present study was first to evaluate the levels of participation in physical activities (PA) and of self-concept in high school and university students, and second to find relationships between these psycho-physical variables. Four hundred and forty adolescents ranging in age from 16 to 20 years, from Spain and Portugal, participated in this study (cross-sectional design). The IPAQ and Self-Concept Form-5 questionnaires were used. The results demonstrated some differences; the Portuguese university students had lower scores in the academic, emotional and physical dimensions and vigorous PA but higher sitting time, walking and moderate PA compared to Portuguese high school students. In addition, Spanish university students had lower self-concept scores although higher practice of PA and lower sitting time than Spanish high school students (*p* < 0.05). On the other hand, a positive relationship was found between different dimensions of self-concept and levels of PA, while the relationship between these dimensions and the time the adolescent spent sitting was negative. In conclusion, the university students have lower scores of self-concept dimensions (in general), and the practice of PA is higher in Spanish university students, confirming the clear presence of differences between both educational stages. The relationship between the dimensions of self-concept in general and the level of PA was positive (following the contributions of Marsh).

## Introduction

The scientific literature has demonstrated that regular practice of moderate (MPA) and vigorous (VPA) physical activities (PA) provides health benefits for different ages (Reiner et al., 2013). Accordingly, the World Health Organization has recommended that adolescents between 15 and 17 years old should practice at least 60 min of MPA and VPA daily, and at later ages, at least 150 min of MPA or 75 min of VPA weekly (World Health Organization, 2010). These ages, which include adolescence as well as the start of adulthood, are particularly important due to their propensity for forming healthy lifestyle habits. For this reason, Hills et al. (2015) recommend programs to create such habits and to reduce physical inactivity, as the latter could lead to the onset of certain health risk factors such as overweight and obesity (Du et al., 2014).

More specifically, adhering to a practice of PA is considered one of the principal solutions to avoid these risk factors and cardiovascular diseases, thereby fostering good health and quality of life (Myers et al., 2002; Blair et al., 2012; Kokkinos, 2012). Nevertheless, adolescents do not regularly practice PA, neither in high school (Olds et al., 2009) nor in university (Pengpid et al., 2015). This lack of practice has become one of the major problems of today’s society in general (World Health Organization, 2010; Kohl et al., 2012), as there exist a significant number of persons who are sedentary as compared to those who are physically active (Expósito et al., 2012).

The beginnings of unhealthy lifestyles seem to appear, predominantly, in the period of adolescence which coincides with the transition from high school to university, where these young adults are subjected to multiple changes because of their psychological instability (Smetana et al., 2006). The transition to university is seen as an exciting period for many students in their first year as it involves new challenges in their lives (Rach and Heinze, 2017) and also greater responsibility, which consequently poses risks for their self-concept (Salinas-Miranda et al., 2015). The changes in healthy lifestyle and habits that occur during this transition are manifested in a lower level of PA (Varela-Mato et al., 2012), in part due to the elimination of required physical education classes (Chen et al., 2014). These, along with a change of city for the majority of students, are factors that may make PA problematic during the university period (Rona and Gokmen, 2005).

The assessment of healthy habits at these ages is particularly important given that these habits could affect overall self-concept. Self-concept is composed of five dimensions: academic, social, emotional, family, and physical. The relationship of these dimensions with the healthy behavior of adolescents can determine self-improvement actions (Marsh, 1989; Pastor et al., 2006) such as taking part in PA and having adequate nutrition, in the pursuit of quality of life (Serra-Majem et al., 2004; Wanden-Berghe et al., 2015). The performance of PA is related to self-concept (Slutzky and Simpkins, 2009), even to the point of possible changes in the student’s personality and his capacity to understand his own behavior (Marsh and Redmayne, 1994), his relationship with others, his deficiencies, and his motivations. Physical self-concept was both an effect and a cause of exercise behavior (Marsh and Martin, 2011). For their part, Cerkez et al. (2015) attest to the fact that regular participation in PA has a positive relation with physical self-concept. When there exist a low level of participation in PA, the person may be vulnerable in his social self-concept due to dissatisfaction with his body image (Annesi, 2010; Chen and Lee, 2013; Zschucke et al., 2013; Owen et al., 2014). On the other hand, VPA can affect the emotional dimension of self-concept during the transition. This dimension measures the perception of a person’s emotions and the degree of control he has over them. It is a significant dimension to investigate in adolescents who are entering university because of the stress and fatigue that VPA, such as the practice of sports, can cause (Strong et al., 2005).

For these reasons, the importance of PA is stressed, as a means by which adolescents can improve their physical condition and competence, body image, self-esteem, and self-concept (Crocker et al., 2000; Moreno et al., 2007; Rojas et al., 2011). Our hypothesis is that the level of self-concept is related positively to the level of participation in PA, expressed as VPA and/or MPA.

Despite the numerous studies of adolescents and university students which look at self-concept and its relation with PA, there are none which analyze if this relationship is different for high school students as compared to university students in order to understand the effect this school transition may have on these young adults. A better understanding of these associations, and the differences found between university and high school students, would improve our current understanding and help in designing intervention strategies focused on increasing PA as a path toward improved self-concept. Accordingly, the present study proposes two objectives. The first objective was to assess the levels of PA and self-concept during the transition from high school to university. The second objective was to analyze relationships between the level of PA and the self-concept dimensions.

## Materials and Methods

### Participants and Procedure

**Figure 1:**
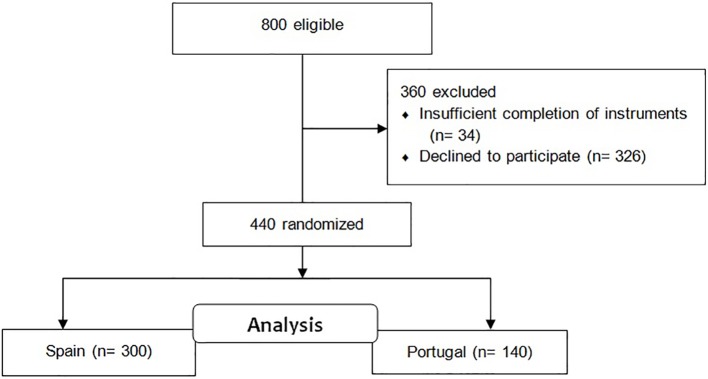
Flow diagram of recruitment of participants.

We selected the school participants by a cluster sampling, taking into account the population from high schools and colleges situated in the South of Spain and in the North of Portugal. Initially, the recruitment of students was carried out through the same protocol in both countries. Twelve high schools (six from Spain and six from Portugal) and eight colleges (four from Spain and four from Portugal) were selected at random. All of them agreed to participate in the study except for one college in Portugal. Subsequently, the web link was sent to 400 high school students (200 from Spain and 200 from Portugal) and 400 university students (200 from Spain and 200 from Portugal). Of the total number of students, 311 from Spain and 163 from Portugal completed it. Finally, 34 participants were eliminated because of insufficient completion of the different instruments, leaving an uncompleted response rate of 7.17% (Figure 1). Our final sample consisted of 300 Spanish students [mean (*M*) age = 17.9, standard deviation (SD) = 3.4 years; weight *M* = 65.1, SD = 12.0 kg; height *M* = 171.2, SD = 9.2 cm] and 140 Portuguese students (age *M* = 18.4, SD = 3.1 years; weight *M* = 64.2, SD = 9.7 kg; height *M* = 170.3, SD = 9.5 cm). The participants were studying from the last two grades of the high school (before the transition) until the first two grades of university (after the transition). Their ages ranged from 16 to 21 years.

A cross-sectional, descriptive, and observational quantitative methodology was used in this study. The parents, in the case of the high school students, were informed of the aims and signed the consent form prior to the participation of the students in this study, and all the participants also gave their written consent to voluntarily participate in the research. The study was approved by the Ethics Committee of the University of Granada (Spain) and followed the guidelines found in the Helsinki Declaration (2013) which establishes ethical principles for investigations using human beings and confidentiality. The authors sent an email invitation to participate in the study to the teachers of the institutions. When we received the acceptance to participate by the teacher, we sent the consent so they could sign it. All participants were measured for weight and height. In the case of high schools, these measurements were carried out by the physical education teacher, and in the colleges, the researchers of this study, always through the same instrument. Once we had the physical and socio-demographic characteristics, they were able to carry out the questionnaires *via* the Internet that were provided through the media of the groups. They received this email invitation that contained the web link to the study with aims and tests of the study. The identification of the student was never requested.

### Instruments

Physical and social-demographic characteristics were collected from the participants through an ad-hoc test with questions regarding age, weight, height, sex, grade, and educational level. Electronic scale SECA (Hamburg, Germany) was used to measure body weight to the nearest 100 g and height was measured to the nearest 0.1 cm with a SECA electronic stadiometer (Seca Ltd., Medical Scales and Measurement Systems, Birmingham, United Kingdom). After that, we could calculate the overweight level of the participants using BMI according to Cole et al. (2000) and World Health Organization (2016) classification system (normal-weight if BMI < 25.0 kg·m^−2^; overweight if BMI ≥ 25.0 kg·m^−2^).

The Self-Concept Questionnaire, called Form 5, was validated for the Spanish sample (García and Musitu, 2001; level of reliability = 0.85), is based on the theoretical model created by Shavelson et al. (1976), and consists of five subscales, each one measured by six items: academic/occupational (items 1, 6, 11, 16, 21, and 26), social (items 2, 7, 12, 17, 22, and 27), emotional (items 3, 8, 13, 18, 23, and 28), family (items 4, 9, 14, 19, 24, and 29), and physical (items 5, 10, 15, 20, 25, and 30). In this way, using a Likert-type scale from 1 to 99, one single instrument measures the principal dimensions of self-concept (Grandmontagne and Fernández, 2004). In this study, the reliability level was *α* = 0.859 (higher than original version with 0.810), where all the dimensions had optimized values (academic, *α* = 0.898; social, *α* = 0.878; emotional, *α* = 0.858; family, *α* = 0.882; physical, *α* = 0.779).

To measure PA levels, participants completed the International Physical Activity Questionnaire-Short Form (IPAQ-SF; Booth, 2000). This questionnaire has been validated in different countries (Craig et al., 2003), and it has shown acceptable psychometric properties to assess PA levels with good reliability, as the Spearman correlation coefficient values are around 0.8. The Spanish IPAQ-SF reliability for measuring PA levels used for both high school and university students has been assessed and verified (*r* = 0.49, *p* < 0.001; Rodriguez-Muñoz et al., 2017). According to the official IPAQ scoring protocol, we calculated total daily PA by summing the product of reported time in PA level and sitting time.

### Statistical Analysis

The influences of the country (Spain and Portugal) and the educational stage (high school and University) factors on academic, social, emotional, family, and physical dimensions of self-concept and PA variables [VPA, MPA, walking activities (WPA), and sitting time] were analyzed using two-way ANOVA after checking the normality and homogeneity assumptions (Pallant, 2011). A Bonferroni correction *post hoc* was performed to make comparisons between groups. Furthermore, an independent samples *t*-test (not paired samples *t*-test) was used for educational stage as an independent variable (using the last grade of high school and the first year at university) after validating normality and homogeneity assumptions (Pallant, 2011). The effect size was presented as *η*^2^ for a two-way ANOVA test and interpreted using the follow criteria: minimum effect (*η*^2^ ≤ 0.02), moderate effect (0.02 < *η*^2^ ≤ 0.09), and strong effect (*η*^2^ > 0.09) (Lakens, 2013). For the case of independent samples *t*-test, Cohen’s *d* was executed as effect size using the follow criteria: small effect (*d* < 0.20), moderate effect (0.20 ≤ *d* < 0.80), and large effect (*d* ≥ 0.80) (O’Donoghue, 2013). Finally, correlational analysis (Pearson’s test) was carried out in order to assess the relationship between the self-concept dimensions and the PA variables. The data analysis was conducted using IBM SPSS (version 24.0) software for Windows and a statistical significance of 5% (*p* < 0.05) was defined.

## Results

**Figure 2:**
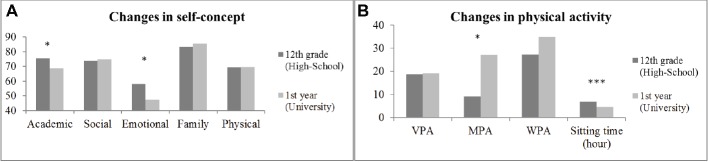
**(A)** Changes in self-concept by grades. **(B)** Changes in physical activity by grades. ^*^p < 0.05; ^***^p < 0.001.

In this section, we consider high school Spanish students (HS), university Spanish students (US), high school Portuguese students (HP), and university Portuguese students (UP). There was significant interaction (Table 1) between country and educational stage for academic self-concept (*F*_(1,440)_ = 5.828; *p* = 0.016; *η*^2^ = 0.013; small effect size); emotional self-concept (*F*_(1,440)_ = 8.879; *p* = 0.003; =0.020; small effect size); physical self-concept (*F* = 26.135; *p* = 0.001; *η*^2^ = 0.057; moderate effect size); VPA (*F*_(1,440)_ = 69.646; *p* = 0.001; *η*^2^ = 0.138; large effect size); MPA (*F*_(1,440)_ = 25.416; *p* = 0.001; *η*^2^ = 0.055; moderate effect size); WPA (*F*_(1,440)_ = 5.26; *p* = 0.022; *η*^2^ = 0.012; small effect size); and sitting time (*F*_(1,440)_ = 28.234; *p* = 0.002; *η*^2^ = 0.061; moderate effect size). In this case, an independent sample *t*-test was used for educational stage, with the observed groups being the last grade of high school and the first year at the university [Figure 2A (self-concept dimensions) and Figure 2B (PA variables)]. The academic and emotional dimensions and the sitting time were lower at university, although the MPA was higher (*p* < 0.05).

**Table 1:** *t*-Test between high school and university students for the self-concept and PA variables, and two-ways ANOVA test (the three final columns) between country and educational stage factors.

		Spain		*p*	*d*	Portugal		*p*	*d*	*F*_(1,440)_	p*	*η*^2^
		High school	University			High school	University					
Academic	(Points)	76.58 ± 15.14^a^	75.43 ± 11.94^a^	0.452	0.082	77.23 ± 7.66	69.23 ± 13.04	0.000	0.734	5.665	0.001	0.038
Social	(Points)	79.51 ± 14.07	78.91 ± 12.22	0.691	0.045	71.37 ± 12.44	74.54 ± 12.43	0.161	0.254	7.247	0.000	0.047
Emotional	(Points)	49.36 ± 24.69^b^	42.98 ± 19.11^b^	0.010	0.280	67.90 ± 6.67	48.34 ± 17.55^b^	0.000	1.434	19.207	0.000	0.117
Family	(Points)	84.76 ± 13.36	86.04 ± 9.93	0.331	0.105	88.65 ± 4.24	86.05 ± 10.11	0.060	0.327	1.839	0.139	0.012
Physical	(Points)	66.36 ± 18.87^c^^,^^b^	74.23 ± 11.02^b^	0.000	0.481	81.43 ± 6.75	72.41 ± 16.05^b^	0.000	0.714	16.529	0.000	0.102
VPA	(min·day^−1^)	22.46 ± 18.53^c^	38.73 ± 13.75	0.000	0.481	24.81 ± 17.72^c^	18.99 ± 17.30^c^	0.068	0.333	71.452	0.001	0.330
MPA	(min·day^−1^)	13.23 ± 12.89^c^	24.77 ± 13.53	0.000	1.426	13.68 ± 7.99^c^	17.11 ± 17.41^c^	0.155	0.247	45.418	0.001	0.238
WPA	(min·day^−1^)	25.25 ± 10.55	30.13 ± 10.71^d^	0.000	1.216	10.75 ± 7.60^c^^,^^d^	18.57 ± 14.23^b^^,^^c^^,^^d^	0.000	0.672	66.651	0.001	0.314
Sitting time	(min·day^−1^)	397.7 ± 208.8	251.2 ± 114.5^d^	0.000	0.975	225.6 ± 88.49^d^	270.7 ± 166.6^d^	0.059	0.332	28.526	0.001	0.164

VPA, Vigorous physical activity; MPA, Moderate physical activity; WPA, Walking physical activity.

* Two-ways ANOVA: significantly different at *p* < 0.05 compared with (Bonferroni *Post hoc*).

a UP (academic self-concept; WPA).

b HP (emotional self-concept; physical self-concept).

c US (physical self-concept; VPA; MPA; WPA).

d HS (WPA; sitting time).

Finally, correlational analysis is shown in Table 2. Bivariate correlation analysis revealed that physical self-concept was positively related to VPA and MPA and negatively related to sitting time both in high school and university (with a greater correlation coefficient for VPA). Furthermore, VPA and MPA were negatively associated with the academic (high school) and emotional self-concept dimensions.

**Table 2:** Correlational analysis.

		Academic	Social	Emotional	Family	Physical
VPA	(High school)	−0.032	−0.020	−0.173^**^	0.059	0.288^**^
	(University)	0.153^*^	0.170^*^	−0.224^**^	0.112	0.204^**^
MPA	(High school)	−0.194^**^	−0.117	−0.160^*^	−0.016	0.129^*^
	(University)	0.029	0.017	−0.111	−0.076	0.019
WPA	(High school)	0.083	0.275^**^	−0.054	0.046	−0.161^*^
	(University)	0.251^**^	0.122	−0.215^**^	0.230^**^	0.126
Sitting time	(High school)	−0.075	0.044	0.082	−0.093	−0.283^**^
	(University)	−0.160^*^	−0.228^**^	−0.301^**^	−0.024	−0.056

^*^p < 0.05; ^**^p < 0.01.

VPA, Vigorous physical activity; MPA, Moderate physical activity; WPA, Walking physical activity.

## Discussion

The purpose of the present study was, first, to evaluate the levels of practice of PA and self-concept in high school and university students, and second, to find relationships between these psycho-physical variables. The results indicated that there are differences of self-concept dimensions and PA variables between countries and educational stages. On the one hand, university Spanish students had lower self-concept scores (small effect size) although higher practice of PA and lower sitting time (large effect size) than high school Spanish students. On the other hand, university Portuguese students had lower self-concept scores (large effect size) and VPA, although higher sitting-time, WPA, and MPA (moderate effect size) than high school Portuguese students. This is in accordance with the scientific literature which shows that this educational transition could suppose important changes in adolescents, especially psychological and emotional changes (Ullrich-French et al., 2013; Akos et al., 2015). The sources of these psychosocial changes are, among others, the appearance of stress, social problems (friends or family), and physical changes characteristic of this age, which all affect personality development. Hagger et al. (2005) indicated that although there were also some statistically significant decreases in self-concept ratings across grade, the effect sizes were small and offered little evidence for developmental changes in physical self-concept. In this study, the effect size was large in both populations, although the tendency in Spanish students was increasing, and in Portuguese students, it was decreasing with respect to physical self-concept.

The university Spanish students had higher practice of PA in general (VPA, MPA, and less time sitting). These results differ from those found in the studies by Cocca et al. (2014) and Sevil et al. (2018), who observed lower scores in the practice of PA in university students, as occurred with the Portuguese students in our study. This could be due to the students’ discipline of studies (science vs. humanities), mode of transport from residence to the campus, and in general, available free time. These changes in lifestyle can provoke personal, emotional, and social changes which may be manifested in tobacco and alcohol consumption, diet, and time spent sitting in front of the television (Romaguera et al., 2011). This, in turn, has the immediate consequence of a reduction in the emotional dimension of self-concept (appearing in all the students) and in the academic dimension (seen in the Portuguese university students), which ends up producing a change in personality (Slutzky and Simpkins, 2009). Nevertheless, the Spanish university students, in comparison with their high school counterparts, reduced their sitting time in favor of increasing their practice of PA.

This study also examined the differences in the existing associations between the psycho-physical variables found in both educational stages (high school and university). In a concrete way, there are several correlations between specific dimensions of self-concept and PA variables. This fact has been studied through various contributions by Marsh (Marsh and Redmayne, 1994; Marsh and Martin, 2011; among others), which confirms the causal existence and effects between this construct and the PA or physical fitness of children and adolescents. However, until now, the differences and relationships of these variables have not been described taking into account the educational transition to university, having knowledge of the contextual variables and evolution that this population has. To start, the academic, social, and family self-concept dimensions were positively related to the level of the practice of PA in accordance with other studies (Annesi, 2006; Esnaola and Revuelta, 2009; Contreras et al., 2010; Bean et al., 2012). Higher levels of practice had a positive effect on the social behavior and health of the adolescents (Esnaola et al., 2018). However, these relationships were seen mostly in the university students. In high school students, only the social and physical dimensions were positively related to WPA and VPA, respectively. A possible explanation could be that the mandatory requirement of physical education classes in high school, as opposed to the voluntary practice of PA by university students for social and health reasons, produces a different effect on self-concept (Roberts et al., 2015). Sevil et al. (2018) conclude that intervention programs are needed to improve sports practice and the dimensions of self-concept in order to maintain or increase the levels of PA during the transition to university for the Portuguese students. On the other hand, the emotional dimension of self-concept was inversely related to VPA in all the students. This may have been due to the anxiety and stress associated with overtraining, or perhaps the type of sport practiced, given that repeated vigorous actions provoke a weakening of mental processes, which ultimately affects the emotional dimension (Strong et al., 2005). Finally, it is necessary to take into account the type of sport practiced, individually or as a team, the duration of the practice, the frequency of sports practice, and adherence. All of these are factors that can affect self-concept to a greater or lesser extent (Berger, 1996).

The present study has some limitations which should be considered in future research, one of which is the limited size of our sample. Even though our study included 440 participants from Spain and Portugal, this number should be increased in order to establish more robust statistical conclusions. Furthermore, the high rate of attrition in the Portuguese sample could be due to lack of interest to participate in the study, since the tests were provided through a web link and thus anonymity was maintained. This problem could have been solved by making some additional reminders or by expanding the sample bias in other universities even if they are more widely spaced. The results demonstrated that the university students had lower self-perceptions than students at the high school, and also it seems to have scarce relation to the practice of PA. Therefore, it is necessary to encourage future longitudinal studies to investigate intra-participant information. In this way, a cause-effect relationship could be confirmed on the generalizable fluctuation of the adolescent’s self-concept. In addition, these results provided could be predetermined by other school stages, which strengthens the idea of carrying out future longitudinal studies. Another future line of research could be to investigate the effect of the university transition on males versus females. It is believed that there are differences to consider and discuss due to academic, emotional, and hormonal changes which occur in both sexes at these ages. Finally, it is possible to broaden the study by evaluating the relationship between levels of obesity and self-concept dimensions before and after the transition to university. The hypothesis would be that since these students have lower self-concept (than their normal-weight counterparts) in the physical, emotional, and social dimensions before the transition, the reduction in self-concept after the transition would possibly be lower.

In conclusion, the main findings of this study indicate that, on the one hand, the academic, emotional, and physical dimensions of self-concept and in the level of PA are lower in university students, admitting certain differences in the two countries studied. On the other hand, there exists a positive relationship between the self-concept dimensions in general and the level of practice of PA, and conversely, a negative relationship between the self-concept dimensions and the amount of time the adolescent spends sitting. In addition, all measures were self-reported and, as such, they could be biased upwards. For this reason, these results and the conclusions of this study should be taken into consideration with caution. Although the results provided correspond to different samples (inter-participant differences) where the independent variable has not been manipulated, we consider that these results could be supported by others whose study belongs to a longitudinal design. These results reinforce the need to promote active lifestyles in adolescents with the objective of enhancing his well-being and development in this stage of his life. The practical application of these findings is to serve as a stimulus for the promotion of the practice of PA during the transition to university, as it is a critical period in the life of adolescents.

## Ethics Statement

The study was approved by the Ethics Committee of the University of Granada (Spain) and followed the guidelines found in the Helsinki Declaration (2013) which establishes ethical principles for investigations using human beings and confidentiality.

## Author Contributions

WO-O and AC-R contributed conceptualization, data curation, methodology, project administration, resources, supervision, validation, writing – original draft and review and editing. JC-M and FM contributed formal analysis, funding acquisition, investigation, methodology, project administration, resources, supervision, visualization, writing – original draft and writing – review and editing.

### Conflict of Interest Statement

The authors declare that the research was conducted in the absence of any commercial or financial relationships that could be construed as a potential conflict of interest.
